# Bivariate binary analysis on composite index of anthropometric failure of under-five children and household wealth-index

**DOI:** 10.1186/s12887-021-02770-5

**Published:** 2021-07-31

**Authors:** Demeke Lakew Workie, Lijalem Melie Tesfaw

**Affiliations:** Department of Statistics, Bahir Dar University, Bahir Dar, Ethiopia

**Keywords:** Bivariate binary logistic regression, Composite index anthropometric failure, Ethiopia, Household wealth index, Under five children.

## Abstract

**Background:**

Malnutrition is the most common cause of mortality and morbidity of children in low and middle income countries including Ethiopia and household wealth index shares the highest contribution. Thus, in this study it is aimed to conduct bivariate binary logistic regression analysis by accounting the possible dependency of child composite index anthropometric failure and household wealth index.

**Methods:**

In this study the data from Ethiopian Demographic and Health Survey (EDHS) 2016 involved 9411 under five children was considered. Child Composite Index Anthropometric Failure (CIAF) measures the aggregate child undernourished derived from the conventional anthropometric indices (stunting, underweight and wasting). The correlation between CIAF and wealth index was checked and significant correlation found. To address the dependency between the two outcome variables bivariate binary logistic regression was used to analyze the determinants of child CAIF and household wealth index jointly.

**Results:**

Study results show that region, place of residence, religion, education level of women and husband/partner, sex of child, source of drinking water, household size and number of under five children in the household, mothers body mass index, multiple birth and anemia level of child had significant association with child CIAF. Female children were 0.82 times less likely to be CIAF compared to male and multiple birth children were more likely to be CIAF compared to single birth. Children from Oromia, Somalie, Gambela, SNNPR, Harari and Addis Ababa region were 0.6, 0.56, 0.67, 0.52, 0.6 and 0.44 times less likely to be CIAF compared to Tigray. A household from rural area were 15.49 times more likely poor compared to a household. The estimated odds of children whose mothers attended primary, and secondary and higher education was 0.82, and 0.52 times respectively the estimated odds of children from mothers who had never attended formal education.

**Conclusion:**

The prevalence of children with composite index anthropometric failure was high and closely tied with the household wealth index. Among the determinants, region, religion, family education level, and anemia level of child were statistically significant determinants of both CIAF and household wealth index. Thus, the authors recommend to concerned bodies and policymakers work on household wealth index to reduce the prevalence of child composite anthropometric failure.

## Background

Malnutrition is a relative or absolute deficiency or excess of one or more essential nutrients, and it is still a crucial problem, in particular, of children aged under five in the world [1]. It is the most common cause of mortality and morbidity, and responsible for more than half of death in children in low and middle income countries such as Ethiopia [2, 3]. Child malnutrition is the major public health problems in the world, with estimate of 45% of all death among under five children [4]. In Africa, the prevalence of chronic malnutrition is about 39% among under five children, and in Ethiopia it is worse higher than other African countries [4], revealed that more than one third of child death is associated with malnutrition [5]. Because, children are most vulnerable to malnutrition due to low dietary intakes, inequitable distribution of food within the household, improper food storage and preparation [6].

Malnutrition is devastating problems, particularly low-economic countries like Ethiopia as half of the Ethiopian population is living below the food poverty line and there is difficulty to meet their daily minimum nutritional requirement [6]. The anthropometric indicators involve wasting (weight-for-height), stunting (height-for-age), and underweight (weight-for-age) are among those commonly used to measure malnutrition in a population of childhood [4, 7]. Children whose measurements fall below 2 standard deviations from the reference median are generally considered malnourished and each indicator captures different aspects of malnutrition [7]. Based on these three indices the total malnourished of children can be detected using composite index of anthropometric failure (CIAF) [8]. In this study the composite index of stunting, wasting and underweight as composite index of anthropometric failure (CIAF) was employed for better description of malnutrition [8, 9]. Despite some improvements and remarkable efforts done to reduce child malnutrition rates, nearly half of under five children in Ethiopia are still malnourished [7].

Several socio-economic, demographic, and biological factors contribute to malnutrition. Of these, household wealth index shares the highest contribution and a key determinant of malnutrition which influence dietary intake and health cares of children and mothers. Household wealth index is an indicator of access to adequate food supplies, use of health services, availability of improved water sources, and sanitation facilities [6]. Several studies in Ethiopia [5–7] showed that higher household wealth index leads to lower levels of child malnutrition and it is indicated that low-income areas need consideration for prioritization and design of interventions to prevent and reduce malnutrition [10]. As researchers’ knowledge concerned, several studies of malnutrition [4, 5, 11], the anthropometric indicator wasting, stunting, and underweight were analyzed separately as malnutrition. However, in this study all the three anthropometric indicators incorporated and valued as Composite Index Anthropometric Failure (CIAF) for better description of malnutrition over other studies. Besides to this, the risk factors of child malnutrition and household wealth index studies conducted using univariate binary analysis, and a little attention is given for bivariate binary analysis in order to assess the association between child malnutrition and household wealth index with effects of socio-economic, demographic and biological factors. Thus, in this study it is aimed to conduct bivariate binary logistic regression analysis on the association between child CIAF and household wealth index relying on other socio-economic, demographic, and biological characteristics of children and households.

## Methods

### Data Source

The data for this analysis was obtained from 2016 Ethiopia Demographic and Health Survey (EDHS). The 2016 EDHS sample was stratified in to urban and rural areas and selected in two stages. In the first and second stages, a total of 645 enumeration areas (EAs) and 28 households per EA were selected with probability proportional to EA size and using systematic sampling, respectively. Based on the household and womens’ questionnaire, the children’s data including its height and weight measures was analyzed from 9411 children aged below 59 months [12].

The household and women’s survey questionnaire are two questionnaires among the five (the Household Questionnaire, the Woman’s Questionnaire, the Men’s Questionnaire, the Biomarker Questionnaire, and the Health Facility Questionnaire) that developed by Ethiopian Central Statistical Agency to reflect the population and health issues relevant to Ethiopia [12].

## Variables

### Dependent Variables

The outcome variables for this study were child composite index anthropometric failure (CIAF) and household wealth index. The first outcome variable, child CIAF measures the aggregate child undernourished derived from the conventional anthropometric indices (stunting, underweight and wasting) that classifies into seven groups (no failure; stunted only; underweight only; wasted only; stunted and underweight; wasted and underweight; wasted, stunted, and underweight) [8, 13]. Stunting, underweight and wasting are defined using Z-score as Z $i=\frac{AI_i-\mu }{\sigma }$, where, AI_*i*_ is the individual (child) anthropometric indicator, and refer respectively to median and standard deviation of the reference [14]. The CIAF is a better index for assessing the overall prevalence of undernourished and for identifying children with multiple anthropometric failures in a population [9, 13, 14]. For this study a single child CIAF was computed and re-coded into binary outcome as:” 1 = yes” if a child is either stunted only; underweight only; wasted only; stunted and underweight; wasted and underweight; or wasted, stunted and underweight; and” 0 = no” if a child is neither of them (no failure) [7].

The second outcome variable, household wealth index was computed according to national level wealth index/quantiles by Ethiopian Statistical Agency from poorest to richest utilizing family unit resource data by means of a principal components’ analysis [12, 15], where the twenty, forty, sixty, eighty and hundred percentiles are assigned to poorest, poorer, medium, rich, and richest. For the purpose of this study, household wealth index was categorized as poor = 1 and rich = 0 by merging poorest and poor as poor, and middle, rich and richest as rich.

### Explanatory Variables

The selection of explanatory variables are theoretically driven that draw support from prior research with regard to factors affecting children’s nutritional status and household wealth index. Previous studies are referenced in creating categories for naturally continuous and discrete variables [16].

## Statistical Analysis

### Ordinary Binary Logistic Regression Model

Ordinary binary logistic regression model is considered only single response variable with binary outcome given other covariates. Let **Y**_*i*_ = (Y_*i*1_,Y_*i*2_) be a vector of binary responses indicating whether the i^*th*^ child is CIAF (Y_*i*1_ = 1) and a household is poor (Y_*i*2_ = 1) while **X**_*i*_ is the vector of covariates for the i^*th*^ child. Then the ordinary binary logistic regression model is given by:

1 $$\mathit{\log} it\; pr\left({y}_{ij}=1\left|{x}_i\right.\right)={\beta}_{1j}{x}_{i1}+{\beta}_{2j}{x}_{i2}+\dots {\beta}_{pj}{x}_{ip}={X}_i{\beta}_j,j=1,2.$$

and the probability that the i^*th*^ child will be malnourished (Y_*i*1_ = 1) or poor household (Y_*i*2_ = 1) is [17]:

2 $$\mathit{\Pr}\left({y}_{ij}=1\right)=\frac{e^{\beta_1{\chi}_{i1}+{\beta}_2{\chi}_{i2}+\dots +{\beta}_p{\chi}_{ip}}}{1+{e}^{\beta_1{\chi}_{i1}+{\beta}_2{\chi}_{i2}+\dots +{\beta}_p{\chi}_{ip}}}=\frac{e^{X_i{\beta}_j}}{1+{e}^{X_i{\beta}_j}}$$

The **X**’s are p-explanatory variables that may affect the outcomes and *β*’s are the coefficients of **X**’s that estimated from the data. The coefficients *β*’s allows researchers to rank the relative significance of explanatory variables and to understand their impact [15].

### Bivariate Binary logistic Regression Model

We can perform separate analysis for the two outcomes CIAF and household wealth index (HHWI) by fitting ordinary binary logistic regression. This, however, would ignore the dependency between the two outcomes. To address this problem and take into account the dependency between two outcomes bivariate logistic regression model was used. Bivariate binary logistic regression analysis is a statistical method used to modeling two binary dependent variables jointly as a function of covariates [18]. In this study, the two outcomes of interests are child CIAF (yes = 1, no = 0) and household wealth index (poor = 1, rich = 0). In case of bivariate binary logistic regression analysis two binary outcomes (Y_1_, Y_2_) has four possible joint probabilities as: 
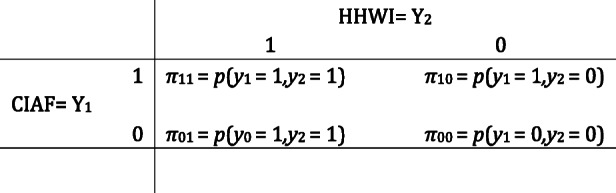


Therefore, the joint probabilities expressed as a function of explanatory variables (**x**_*i*_) is given by [18]:

3 $${\pi}_{11}\left({\chi}_i\right)=p\left({y}_1=1,{y}_2=1\left|{\chi}_i\right.\right)=\frac{e^{X_i{\beta}_{11}}}{1+{e}^{X_i{\beta}_{11}}},\kern0.5em child\kern0.5em is\kern0.5em CIAF\kern0.5em and\kern0.5em from\kern0.5em poor\kern0.5em household$$

4 $${\pi}_{10}\left({\chi}_i\right)=p\left({y}_1=1,{y}_2=0\left|{\chi}_i\right.\right)=\frac{e^{X_i{\beta}_{10}}}{1+{e}^{X_i\beta 10}},\kern0.5em child\kern0.5em is\kern0.5em CIAF\kern0.5em and\kern0.5em from\kern0.5em rich\kern0.5em household$$

Alternatively, these can be articulated as logit function as:

5 $$logit\left[{\pi}_{11i}{X}_i\right]={X}_i{\beta}_{11} and\ logit\left[{\pi}_{10i}{X}_i\right]={X}_i{\beta}_{10}$$

In the Eq. (3, 4, 5) *β*_11_ and *β*_10_ refers to the parameters of the conditional logit model for *Y*_2_ = 1 and *Y*_2_ = 0, respectively in a function of predictors, **X**. These parameters used to express the nature of dependency between the outcome variables *Y*_1_ and *Y*_2_ in the presence of explanatory variables. A regression model for the odds ratio (OR) [18], which describes the dependence of child CIAF and household wealth index is fitted as:

6 $${OR}_i=\frac{\pi_{11i}\left({X}_i\right)/\left[1={\pi}_{11i}\left({X}_i\right)\right]}{\pi_{10i}\left({X}_i\right)/\left[1={\pi}_{10i}\left({X}_i\right)\right]}=\frac{e^{X_i{\beta}_{11}}}{e^{X_i{\beta}_{10}}}={e}^{\left({\beta}_{11}-{\beta}_{10}\right){X}_i} and\kern0.5em \mathit{\log}\kern0.5em \left({OR}_i\right)={X}_i\left({\beta}_{11}-{\beta}_{10}\right)$$

The dependence between Y_1_ and Y_2_, can measure in terms of odds ratio and any departure from odds ratio = 1 will indicate the extent of dependence. The null hypothesis *H*_0_: *β*_11_ = *β*_10_ can be tested for independence in the presence of covariates between the two binary outcome variables Y_1_ and Y_2_.

### Parameter Estimation and Model Diagnosis

All the logistic models of the parameters are estimated by the maximum likelihood techniques using Newton-Raphson algorithm iterative procedure and individual parameter estimates are tested based on the *H*_0:_ *β*_1*j*_ = 0 versus *H*_1:_ *β*_1*j*_ 6 = 0 using the Wald statistic $W=\frac{{\hat{\beta}}_{1 ji}}{\hat{s}e\left({\hat{\beta}}_{1 ji}\right)}$ [17]. The goodness-of-fit test of the model was checked using correct classification rate (CCR). CCR is the ratio of number of correct predictions to number of observations. When the number of correct predictions is high, the CCR is high which indicates that higher accuracy and hence, the estimated model is good fit of the data [17].

The data was coded and entered to Statistical package for social science (SPSS) version 26 for Windows. The SPSS version 26 and R version 4.0.0 with VGLM package were used for the data analysis [19].

## Results

### Exploratory Analysis

In this study, a total of 9411 under five children were involved. The exploratory analysis was presented in four tables. Tables 1 and 2 revealed the description and frequency of dependent and independent variables, respectively. Nearly half of the children involved in this study had CIAF (46.7%) and from poor household (46.4%) (Table 1).

**Table 1:** Dependent variables description and its frequency

Dependent variables	Categories (Codes)	n (%)
CIAF	Yes (1) No(0)	4393 (46.7%) 5018 (53.3%)
Wealth index	poor (1) rich (0)	4371 (46.4%) 5040 (53.6%)

**Table 2:** Explanatory variable description and frequency

Variables	Categories (Codes)	n (%)	Variables	Categories (Codes)	n (%)
Age of child	0–5 (0)	1047 (11.1)	Mother’s education	No education (0)	6194 (65.8)
	6–11 (1)	953 (10.1)	Level	Primary (1)	2577 (27.4)
	12–23 (2)	1809 (19.2)		Secondary and above (2)	641 (6.8)
	24–35 (3)	1884 (20.0)			
	36–47 (4)	1848 (19.6)	Age of mothers	*<* 20 (0)	6113 (65.0)
	48–59 (5)	1870 (19.9)	at first birth	20–34 (1)	3288 (34.9)
				35–49 (2)	10 (0.1)
Sex of child	Male (1)	4816(51.2)			
	Female (2)	4595(48.8)	Mothers BMI	Thin (0)	1829 (19.4)
				Normal (1)	6877 (73.1)
Region	Tigray (1)	643(6.8)		Overweight (2)	615 (6.5)
	Afar (2)	90 (1.0)			
	Amhara (3)	1824 (19.4)	Anemia	Non Anemic (0)	4722 (50.2)
	Oromia (4)	4145 (44.0)		Anemic (1)	3511 (37.3)
	Somali (5)	391 (4.2)		Missing	1179 (12.5)
	Benishangul (6)	100 (1.1)			
	SNNPR (7)	1935 (20.6)	Cough	No (0)	7512(79.8)
	Gambela (8)	22 (0.2)		Yes (1)	1900 (20.2)
	Harari (9)	20 (0.2)			
	Addis Adaba (10)	206 (2.2)	Diarrhea	No (0)	8274 (87.9)
	Dire Dawa (11)	36 (0.4)		Yes (1)	1120 (11.9)
				Missing	18 (0.2)
Residence	Urban (1)	1022 (10.9)			
	Rural (2)	8389 (89.1)	Fever	No (0)	8021 (85.2)
				Yes (1)	1381 (14.7)
Birth oder	1st (0)	1700(18.1)		Missing	10 (0.1)
	2–3 (1)	2895 (30.8)			
	4–5 (2)	2263 (24.0)	Husband/partner’s	No education (0)	4232 (45.0)
	6 and more (3)	2553 (27.1)	education level	Primary (1)	3589 (38.1)
				Secondary and above (2)	1050 (11.2)
Number of children	1(0)	3423 (36.4)		Missing	540 (5.7)
under-five years	2 (1)	2651(27.9)			
in the household	3 or more (2)	1670 (17.7)		1–4 (small) (0)	2411 (25.6)
			Household size	5–9 (medium (1)	6450 (68.5)
Source of drinking	unimproved source (0)	4207 (44.7)		10 and more (Large) (2)	550 (5.8)
water	Improved source (1)	5204(55.3%)	Religion	Orthodox (1)	3253(34.6)
Multiple birth	Single birth (0)	9193 (97.7)		Protestant (2)	2032(21.6)
	1st of multiple (1)	117 (1.2)		Muslim (3)	3842(40.8)
	2nd of multiple (2)	102 (1.1)		Others (4)	285(3.0)

Twenty percent of children was between aged 24–35 months, 51.2% of the children were males and 89.1% lived in rural areas. The majority of mothers (65.0%) were aged less than 20 and 44.7% of households were used unimproved drinking water. Mothers and their parents who didn’t attend education were 65.8 and 45.0% respectively. Nearly one fifth (20.2%), one tenth (11.9%), and one seventh (14.7%) of children had cough, diarrhea, and fever symptoms two months before the survey respectively and more than one third of children (37.3%) have anemia (Table 2).

Table 3 shows the association of covariates with CIAF and household wealth index using chi-square (*χ*^2^) test. The CIAF was statistically associated with age of child, sex of child, region, place of residence, birth order, number of under five children in the household, source of drinking water of the household, multiple birth, age mother at first birth, mothers body mass index, anemia complication, diarrhea and fever symptoms, and husbands and mothers education level as they have *p*-value less than 0.05.

**Table 3:** The association of covariates with child composite index anthropometric failure (CIAF) and household wealth index (HHWI)

Covariates	childCIAF		*p*-value	HHWI		*p*-value
	yes(n%)	no(%)		poor(%)	rich(%)	
Age of child 0–5	455(10.4)	592(11.7)		464(10.6)	583(11.6)	
6–11	407(9.3)	545(10.8)		436(9.9)	516(10.3)	
12–23	840(19.2)	969(19.2)		888(20.2)	921(18.4)	
24–35	924(21.1)	961(19.1)	0.001	862(19.6)	1022(20.4)	0.132
36–47	908(20.8)	940(18.7)		851(19.4)	997(19.9)	
48–59	837(19.1)	1033(20.5)		891(20.3)	980(19.5)	
Sex of child male female	2370(54.2) 2001(45.8)	2446(48.5) 2594(51.5)	0.000	2257(51.4) 2136(48.6)	2560(51.0) 2459(49.0)	0.367
Region Tigray	306(7.0)	337(6.7)		326(7.4)	318(6.3)	
Afar	48(1.1)	41(0.8)		73(1.7)	17(0.3)	
Amhara	993(22.7)	831(16.5)		824(18.8)	1001(19.9)	
Oromia	1875(42.9)	2270(45.0)		1935(44.0)	2210(44.0)	
Somalia	180(4.1)	211(4.2)		301(6.9)	91(1.8)	
Benshangul	50(1.1)	49(1.0)	0.000	60(1.4)	40(0.8)	0.000
SNNPR	846(19.4)	1089(21.6)		842(19.2)	1093(21.8)	
Gambela	8(0.2)	14(0.3)		11(0.3)	11(0.2)	
Harari	8(0.2)	12(0.2)		7(0.2)	13(0.3)	
Addis Ababa	38(0.9)	167(3.3)		0(0.0)	206(4.1)	
Dire Dawa	18(0.4)	18(0.4)		15(0.3)	20(0.4)	
Residence Urban Rural	340(7.8) 4032(92.2)	682(13.5) 4358(86.5)	0.000	77(1.8) 4316(98.2)	945(18.8) 4073(81.2)	0.000
Birth order 1st 2–3 4–5	742(17.0) 1281(29.3) 1086(24.8)	958(19.0) 1615(32.0) 1177(23.4)	0.000	668(15.2) 1319(30.0) 1116(25.4)	1032(20.6) 1576(31.4) 1147(22.9)	0.000
6 and more	1263(28.9)	1290(25.6)		1290(29.4)	1263(25.2)	
No. of children(*<* 5 yrs) 1	1523(34.8)	1900(37.7)		1254(28.5)	2169(43.2)	
2	2083(47.6)	2236(44.4)	0.004	2239(51.0)	2079(41.4)	0.000
3 or more	766(17.5)	904(17.9)		900(20.5)	770(15.3)	
Source of drinking water unimproved source Improved source	2075(47.5) 2296(52.5)	2132(42.3) 2908(57.7)	0.000	2714(61.8) 1679(38.2)	1493(29.7) 3526(70.3)	0.000
Multiple birth Single birth	4234(98.4)	4958(96.9)		4303(98.0)	4889(97.4)	
1st of multiple	71(1.6)	46(0.9)	0.000	48(1.1)	69(1.4)	0.245
2nd of multiple	66(1.5)	36(0.7)		42(1.0)	60(1.2)	
Age of mother(at 1st birth) *<* 20	2925(66.9)	3188(63.3)		2987(68.0)	3126(62.3)	
20–34	1444(33.0)	1844(36.6)	0.001	1404(32.0)	1885(37.6)	0.000
35–49	3(0.1)	7(0.1)		3(0.1)	8(0.2)	
Mothers BMI Thin	935(21.6)	894(17.9)		962(22.2)	867(17.4)	
Normal	3217(74.3)	3659(73.3)	0.000	3208(73.9)	3669(73.7)	0.000
Overweight	176(4.1)	439(8.8)		170(3.9)	445(8.9)	
Anemia Non Anemic Anemic	1514(37.8) 2488(62.2)	1997(47.2) 2233(52.8)	0.000	1433(37.2) 2419(62.8)	2078(47.4) 2303(52.6)	0.000
Cough No Yes	3458(79.1) 913(20.9)	4053(80.4) 987(19.6)	0.291	3573(81.3) 820(18.7)	3938(78.5) 1080(21.5)	0.003
Diarrhea No Yes	3812(87.3) 555(12.7)	4462(88.8) 565(11.2)	0.015	3888(88.7) 493(11.3)	4386(87.5) 627(12.5)	0.033
Fever No Yes	3660(83.8) 710(16.2)	4361(86.6) 672(13.4)	0.000	3809(86.7) 582(13.3)	4211(84.0) 800(16.0)	0.000
Husband/partner education level No education	2175(53.0)	2057(43.1)		2524(61.1)	1708(36.0)	
Primary	1570(38.3)	2019(42.3)	0.000	1460(35.4)	2129(44.9)	0.000
Secondary and above	358(8.7)	692(14.5)		145(3.5)	905(19.1)	
Mothers education level No education	3094(70.8)	3099(61.5)		3428(78.0)	2766(55.1)	
Primary	1113(25.5)	1465(29.1)	0.000	921(21.0)	1657(33.0)	0.000
Secondary and above	165(3.8)	476(9.4)		45(1.0)	596(11.9)	
Household size 1–4 (small)	1075(24.6)	1337(26.5)		1055(24.0)	1356(27.0)	
5–9 (medium)	3034(69.4)	3416(67.8)	0.076	3105(70.7)	3344(66.6)	0.000
10 and more (large)	263(6.0)	287(5.7)		233(5.3)	318(6.3)	
Religion Orthodox	1521(34.8)	1732(34.4)		1334(30.4)	1919(38.2)	
Protestant	897(20.5)	1135(22.5)	0.008	788(17.9)	1245(24.8)	0.000
Muslim	1799(41.1)	2043(40.5)		2090(47.6)	1752(34.9)	
Others	155(3.5)	130(2.6)		182(4.1)	103(2.1)	

The maximum prevalence of CIAF for children under 5 years of age is observed within the age groups of 24 to 47 months. As mothers’ age at first birth increases the prevalence of child CIAF fairly decreases. Whereas the prevalence of child CIAF decreases as educational level of mothers and their parents increase. For instance, the prevalence of anthropometric failure for children under 5 years of age whose mothers had never attended formal education, attended primary, and secondary and above education were 70.8, 25.5, and 8.7% respectively. Nearly half of child CIAF (47.8%) was from households who had two children under five years of age. Compared to child lived in urban area, child CIAF lived in rural area was higher (7.8% vs.92.2%). The prevalence of child CIAF and poor household was vary from region to region. For instance, the highest prevalence of CIAF (42.9%) and poor household (44.0%) was found in Oromia region whereas, the smallest prevalence of CIAF(0.2%) was found in Harari and Gambela region, and poor household(0.0%) was found in Addis Ababa. The prevalence of poor household was fairy decreases as the education level of mothers and their parents increases. That is, the prevalence of poor household with mothers who didn’t attend formal education, attended primary and secondary education and higher was 78.0, 21.0 and 1.0% respectively while the prevalence of poor household with parents had not education, attend primary school and secondary education and higher was 61.1, 35.4 and 3.5% respectively (Table 3).

Table 4 revealed the frequency distribution of each covariate over child CIAF conditioned on household wealth index. The proportion of child CIAF from poor household was equal with child CIAF from rich households at early age of child while child CIAF from poor household were higher compared to child CIAF from rich households and both were increased between age 6 and 35 months. Whereas, as the education level of mothers and their parents increase the child CIAF from poor household was decreased rapidly. The prevalence of male CIAF from poor household was higher compared to female CIAF from poor household (53.1% vs. 46.9%). Similarly, the proportion of child CIAF from poor household who used unimproved drinking water was higher compared to poor household who used improved drinking water (62.2% vs. 37.8%).

**Table 4:** Frequency distribution of covariates for different combinations of child CAIF and HHWI

Covariates	No CAIF	No CAIF	CAIF	CAIF
	and rich (%)	and poor (%)	and rich (%)	and poor
Age of child 0–5	356(12.0)	235(11.4)	225(11.0)	230(9.9)
6–11	326(10.9)	219(10.6)	190(9.3)	217(10.6)
12–23	540(18.1)	429(20.8)	381(18.7)	460(19.7)
24–35	598(20.1)	363(17.6)	425(20.8)	499(21.4)
36–47	573(19.2)	367(17.8)	424(20.8)	484(20.8)
48–59	585(19.6)	449(21.8)	395(19.4)	441(18.9)
Sex of child male	1427(47.9)	1019(49.4)	1132(55.5)	1238(53.1)
female	1551(52.1)	1043(50.6)	908(44.5)	1093(46.9)
Region Tigray	181(6.1)	156(7.6)	137(6.7)	169(7.3)
Afar	9(0.3)	33(1.6)	8(0.4)	41(1.8)
Amhara	520(17.5)	311(15.1)	481(23.6)	512(22.0)
Oromia	1349(45.3)	921(44.7)	861(42.2)	1014(43.5)
Somalia	57(1.9)	154(7.5)	33(16.2)	147(6.3)
Benshangul	24(0.8)	26(1.3)	16(0.8)	35(1.5)
SNNPR	643(21.6)	445(21.6)	450(22.1)	396(17.0)
Gambela	7(0.2)	7(0.3)	3(0.1)	5(0.2)
Harari	8(0.3)	3(0.1)	4(0.2)	4(0.2)
Addis Ababa	167(5.6)	0(0.0)	38(18.6)	0(0.0)
Dire Dawa	12(0.4)	6(0.3)	8(0.4)	10(0.4)
Residence Urban	657(22.1)	25(1.2)	288(14.1)	52(2.2)
Rural	2321(77.9)	2037(98.8)	1753(84.9)	2279(97.8)
Birth order 1st	652(21.9)	306(14.8)	380(18.6)	362(15.5)
2–3	976(32.8)	638(30.9)	600(29.4)	681(29.2)
4–5	656(22.0)	521(25.3)	491(24.1)	595(25.5)
6 and more	693(23.3)	597(29.0)	569(27.9)	693(29.7)
No. of children(*<* 5 years) 1	1333(44.7)	567(27.5)	836(41.0)	687(29.5)
2	1192(40.0)	1044(50.6)	887(43.5)	1196(51.3)
3 or more	453(15.2)	451(21.9)	317(15.5)	449(19.3)
Source of drinking water unimproved source	868(29.1)	1264(61.3)	625(30.6)	1451(62.2)
Improved source	2110(70.9)	798(38.7)	1416(69.4)	881(37.8)
Multiple birth Single birth	2915(97.9)	2043(99.1)	1974(96.8)	2260(97.0)
1st of multiple	38(1.3)	8(0.4)	31(1.5)	40(1.7)
2nd of multiple	25(0.8)	11(0.5)	35(1.7)	31(1.3)
Age of mother (at 1st birth) *<* 20	1845(30.2)	1343(45.1)	1280(62.7)	1644(70.5)
20–34	1126(34.2)	718(24.1)	759(37.2)	685(29.4)
35–49	6(0.2)	1(0.05)	1(0.04)	2(0.08)
Mothers BMI Thin	488(16.4)	406(19.7)	379(18.6)	556(23.9)
Normal	2113(71.0)	1546(75.0)	1156(56.7)	1662(71.3)
Overweight	353(11.9)	86(4.2)	92(4.5)	84(3.6)
Anemia Non Anemic	1305(51.4)	692(40.9)	773(41.9)	741(34.3)
Anemic	1232(48.6)	1002(59.1)	1072(58.1)	1417(65.7)
Cough No	2358(79.2)	1695(82.2)	1580(77.5)	1879(80.6)
Yes	620(20.8)	367(17.8)	460(22.5)	453(19.4)
Diarrhea No	2615(88.0)	1847(89.9)	1771(86.8)	2041(87.7)
Yes	358(12.0)	207(10.1)	269(13.2)	285(12.3)
Fever No	2542(85.6)	1819(88.2)	1670(81.9)	1990(85.4)
Yes	429(14.4)	243(11.8)	370(18.1)	339(14.6)
Husband/partner education No education	914(32.4)	1144(58.8)	794(41.4)	1381(63.2)
Primary	1292(43.4)	727(37.4)	837(43.6)	734(33.6)
Secondary and above	618(21.9)	75(3.9)	288(15.0)	70(3.2)
Mothers education level No education	1530(51.4)	1569(76.1)	1235(60.5)	1859(79.8)
Primary	1009(33.9)	456(22.1)	648(31.8)	465(19.9)
Secondary and above	439(14.7)	37(1.8)	157(7.7)	8(0.3)
Household size 1–4 (small)	855(28.7)	482(23.4)	502(24.6)	573(24.6)
5–9 (medium)	1941(65.2)	1474(71.5)	1403(68.8)	1631(70.0)
10 and more (large)	182(6.1)	106(5.1)	136(6.7)	127(5.4)
Religion Orthodox	1136(38.1)	596(20.9)	783(38.4)	738(31.7)
Protestant	756(25.3)	379(12.7)	488(23.9)	409(17.5)
Muslim	1024(34.4)	1019(49.4)	727(35.6)	1071(45.9)
Others	62(2.1)	68(3.3)	41(2.1)	114(4.9)

Key: *SNNPR* South Nations Nationalities and Peoples Representatives

Table 5 shows the joint and marginal probabilities of child CIAF and household wealth index (HHWI) as well as the odds ratio. The odds ratio (OR) is a natural measure used to describe the association between the two binary responses and a value of unity denotes statistical independence [19, 20]. The OR is differed from unity (1.649). Thus, to fit the joint probability of child CIAF and household wealth index as a set of covariates while accounting for the possible dependency between the two responses using bivariate binary logistic regression model is very natural. Therefore, the bivariate logistic regression analysis of CIAF and household wealth index with other covariates was presented in Table 6.

**Table 5:** Joint and marginal probability of child CIAF and HHWI

		HHWI		Marginal of CIAF	Odds Ratio
		poor = 1	rich = 0		
CIAF	Yes = 1 No = 0	*2331 (0.248)* *2062 (0.219)*	*2040 (0.217)* *2978 (0.316)*	4371 (0.464) 5040 (0.536)	1.469
	Marginal of HHWI	4393 (0.467)	5018 (0.533)	9411 (1.00)	

Key: Numbers in each cell is a frequency and its probability is in the parenthesis

**Table 6:** Parameter estimation of bivariate binary logistic regression modeling of child CIAF and HHWI

Variables	CIAF		HHWI	
	est.(se)	OR (95% CI)	est.(se)	OR (95% CI)
Intercept	0.13(0.18)	1.14(0.80,1.61)	1.08(0.23)	0.34(0.22,0.53)
Region Tigray (*) Afar	0.23(0.15)	0.80(0.60,1.06)	1.06(0.21)	2.89(1.93,4.32)
Amhara	0.18(0.11)	1.19(0.96,1.49)	0.94(0.13)	0.39(0.31,0.50)
Oromia	0.51(0.12)	0.60(0.47,0.76)	1.48(0.14)	0.23(0.17,0.30)
Somalie	0.57(0.14)	0.56(0.42,0.74)	0.35(0.18)	1.41(0.99,0.20)
Benshangul	0.034(0.13)	0.97(0.75,1.25)	0.22(0.15)	0.80(0.60,1.07)
Gambela	0.41(0.13)	0.67(0.52,0.86)	1.20(0.15)	0.30(0.22,0.40)
SNNPR	0.66(0.16)	0.52(0.38,0.71)	1.01(0.20)	2.73(1.86,4.02)
Harari	0.50(0.16)	0.60(0.44,0.83)	1.40(0.20)	0.25(0.17,0.37)
Addis Ababa	0.82(6.37)	0.44(0.01,0.78)	55.34(4460)	0.01(0.00,2.50)
Dire Dawa	0.21(0.17)	0.81(0.58,1.12)	0.25(0.21)	0.78(0.51,1.18)
Residence Urban (*) Rural	0.08(0.09)	1.08(0.91,1.29)	2.74(0.137)	15.49(11.80,20.30)
Religion Orthodox (*) Protestant	0.26(0.11)	1.30(1.05,1.60)	0.53(0.12)	1.70(1.34,2.17)
Muslim	0.33(0.09)	1.38(1.15,1.66)	0.61(0.11)	1.83(1.48,2.26)
Catholic and others	0.51(0.18)	1.67(1.17,2.38)	1.33(0.23)	3.78(2.42,5.90)
Education level of women No education (*) Primary	0.20(0.07)	0.82(0.72,0.94)	0.48(0.08)	0.62(0.53,0.72)
Secondary and higher	0.66(0.13)	0.52(0.40,0.67)	1.11(0.18)	0.33(0.23,0.47)
Diarrhea No (*) Yes	0.10(0.08)	1.11(0.94,1.30)	0.02(0.10)	0.98(0.81,1.19)
Fever No (*) Yes	0.11(0.09)	1.11(0.94,1.32)	0.02(0.11)	0.98(0.79,1.21)
Cough No (*) Yes	0.01(0.08)	1.00(0.85,1.17)	0.10(0.10)	1.11(0.91,1.34)
Multiple birth Single birth (*) First of multiple	0.47(0.24)	1.59(1.01,2.53)	0.13(0.28)	0.88(0.51,1.51)
Second of multiple	0.49(0.25)	1.63(1.00,2.66)	0.07(0.31)	0.93(0.51,1.70)
Sex of Child male (*) female	0.16(0.05)	0.85(0.77,0.94)	0.01(0.06)	0.99(0.88,1.12)
Education level of husband No education (*) Primary	0.19(0.06)	0.83(0.73, 0.93)	0.62(0.07)	0.54(0.47,0.62)
Secondary and higher	0.11(0.09)	0.89(0.75, 1.07)	1.03(0.12)	0.36(0.29,0.45)
Source of drinking water Not improved (*) Improved	0.04(0.06)	0.96(0.86, 1.07)	1.00(0.07)	0.37(0.32,0.42)
Birth order 1st (*) 2–3	0.14(0.08)	0.87(0.74, 1.02)	0.22(0.10)	0.80(0.66,0.98)
4–5	0.06(0.10)	0.95(0.78, 1.14)	0.16(0.12)	0.85(0.67,1.07)
6 and more	0.07(0.10)	0.94(0.77, 1.14)	0.05(0.12)	0.95(0.75,1.21)
Age of mother at first birth less than 20 (*) between 20 and 24	0.11(0.05)	0.90(0.81,1.00)	0.05(0.07)	0.95(0.83,1.08)
between 35 and 49	0.45(1.06)	1.57(0.20,2.60)	1.56(2.30)	4.74(0.05,2.90)
Household size small (1–4) (*) medium (5–9)	0.06(0.08)	1.06(0.91,1.23)	0.31(0.09)	0.73(0.61,0.88)
large (10 and more)	0.18(0.14)	1.20(0.92,1.56)	0.65(0.16)	0.52(0.38,0.72)
Body mass index Thin (*) Normal	0.24(0.06)	0.78(0.70,0.88)	0.20(0.07)	0.82(0.71,1.00)
Overweight	0.80(0.12)	0.45(0.36,0.56)	0.69(0.15)	0.50(0.37,0.67)
Anemia No (*) Yes	0.37(0.05)	1.44(1.30,1.60)	0.37(0.07)	1.44(1.27,1.64)
No. of under five children 1 (*) 2	0.19(0.06)	1.21(1.07,1.36)	0.44(0.07)	1.55(1.35,1.79)
3 and more	0.15(0.08)	0.86(0.73,1.01)	0.43(0.10)	1.54(1.26,1.87)
Age of child 0–5 (*) 6–11	0.01(0.11)	1.00(0.80,1.23)	0.04(0.13)	1.04(0.81,1.35)
12–23	0.15(0.10)	1.16(0.96,1.41)	0.26(0.12)	1.29(1.03,1.62)
24–35	0.17(0.10)	1.18(0.98,1.43)	0.05(0.12)	1.05(0.84,1.32)
36–47	0.18(0.10)	1.20(0.99,1.45)	0.16(0.12)	1.17(0.93,1.47)
48–59	0.05(0.10)	1.05(0.87,1.27)	0.23(0.12)	1.25(1.00,1.58)
**Measure of Dependency** Odds Ratio (OR)		1.498		

Key: *= reference category; *OR* Odds ratio, *est.* estimate, *se* standard error, *CI* Confidence Interval, *SNNPR* South Nations Nationalities and Peoples Representatives

## Parameter Estimates

Table 6 revealed that the effect of covariates on child CAIF and household wealth index by taking into account the dependency of child CIAF and household wealth index using bivariate binary logistic regression model. The dependency between child CIAF and household wealth index was measured using odds ratio (OR) equals 1.498 and a value differ from one indicates significant dependency between child CIAF and household wealth index. After the dependency between child CIAF and household wealth index checked the effect of each covariate on CAIF and household wealth index was identified. Among covariates region, religion, education level of women and husband, sex of child, number of under five children, mothers body mass index, multiple birth, birth order, and anemia level of children were found as significantly associated factors for child CIAF. Whereas, region, place of residence, religion, education level of women and husband, household size, source of drinking water, anemia level of children e and number of under five children had significant association with household wealth index.

Table 7 revealed the predicted and observed counts for a combination of CIAF and household wealth index based on the estimated bivariate binary logistic regression model. The ratio of the number of correct predictions to number of observation, i.e. the correct classification rate (CCR) equals to 80.0% which is very high. This indicates that the estimated model was a good fit of the data.

**Table 7:** Classification of predicted counts based on estimated bivariate binary logistic regression model of child CIAF and HHWI

		HHWI			
		rich		poor	
		observed	predicted	observed	predicted
CAIF	Yes	2363	2030	(1100)	(3420)
	No	3010	(2060)	2008	(2831)

## Discussion

In this study, attempt was made to demonstrate the effects of children and households’ characteristics on children composite index anthropometric failure (CIAF) by considering its dependency with household wealth index (HHWI). A single CIAF was computed from the three classical anthropometric indices and recoded into binary outcome as they have a multidimensional nature [14]. Bivariate binary logistic regression model was employed to determine the effects of covariates on CIAF and HHWI. Based on this model and exploratory data analysis, significant dependency between CIAF and HHWI was noticed. This study demonstrated that child CIAF has significant association with household wealth index given other children’s and households’ characteristics.

This study reveals that the prevalence of child CIAF in Ethiopia was high (46.7%) and which has been also discussed in previous studies [3, 5, 14, 21]. This study found that region in Ethiopia has significant effect on child CIAF and household wealth index. The risk of child CIAF in Tigray was higher than Oromia (OR = 0.60), Somalie (OR = 56), Gambela (OR = 0.67), SNNPR (OR = 0.52), Hararie (OR = 0.60) and Addis Ababa (OR = 0.44). This finding of the study was consistent with different domestic research [5, 22], that reported children who are living in Tigray region have a higher risk of child CIAF compared to children living in Oromia, Somalie, Gambela, SNNPR, Harari, and Addis Ababa. Place of residence has significantly associated with household wealth index. Studies done by Tekile et al., Talukder, and Silveira et al. [4, 5, 21] states that compared to children from middle class and rich households, the likelihood of switching status from malnourished to nourished nutritional status was lower for children from poor household. This indicates that children who were living in rural area is more likely to be CIAF than children living in urban area. This is because of limited infrastructure that enables to affect a family to access food, health-care facility, exchange desired commodities, and so on. This finding was also in line with studies done in Bangladesh and Ethiopia [4].

As education level of mothers and husbands/partners getting higher, the likelihood of child exposed to CIAF was lower. Children whose mothers and husband/partners had never attended education were more likely to exposed to CIAF compared to children whose mothers and husband/partners had primary, and secondary and higher education which was consistent with studies conducted in Ethiopia [5, 22]. These studies reported that children whose mothers had never attended education were significantly more likely to be stunted, underweight, and/or under nutrition as compared to children whose mothers had primary, and secondary and above education level. Moreover, religion of the household was also an important determinate that significantly associated with child CIAF.

The result showed that a child whose household religion orthodox was less likely to be CIAF and more likely to be rich compared to its counterpart. Whereas the reality in Ethiopia shows that the majority Muslim and protestant households were lived in urban area whereas the majority orthodox households are living in rural area where health care facilities, child feeding practice, access to improved water, and so on are less accessible and poverty is very high (OR for urban versus rural is 15.49). Mostly mothers in rural area are participated in farming activities and giving less attention to feed and care of their child. Beyond to this, orthodox mothers are commonly fasting during their prenatal and postnatal period. Therefore, the pre-specified and other issues may be responsible for child CIAF whose household beliefs orthodox than other religious in contrast of this study. In addition, the household who drank improved water were more likely to be rich compared to the household who drank unimproved water.

The existence of CIAF was significantly differ within sex of child and multiple of birth i.e., males were more likely to be CIAF compared to females and multiple birth children were more likely to be CIAF compared to single birth. This result is in line with studies done at Ethiopia and Favelas Brazil [5, 11]. On the other hand, compared to children who drank unimproved water, children who drank improved water were less likely to belonging in poor household and this led to less likely to be CIAF, which is supported by previous study findings [11, 14]. Like a study done in Ethiopia [5], number of under five children in the household was an important determinant effect on child CIAF. Children from a household having two under five children were 1.21 times more likely to be CIAF compared to a household having one under five children.

Unlike a study in Ethiopia and Brazil [5, 11], mothers age at first birth and child age were insignificantly associated with child CIAF in this study. This may because of the methodology difference, i.e., in this study a bivariate binary logistic regression accounting the dependency of a single CIAF and household wealth index was employed whereas the previous studies were used binary and ordinal univariate logistic regression.

Mothers’ body mass index was significantly associated with child CIAF which in line with a study done by Silveira et al. [21] and they stated that short height and poor weight of mothers were associated with child malnutrition. Moreover, this study found that anemic children were more exposed to CIAF compared to non-anemic children that in line with a study done in Ethiopia [22].

## Conclusion

The prevalence of children with composite index anthropometric failure was high and closely tied with the household wealth index. Among covariates region, religion, residence, education level of women and husband, sex of child, number of under five children, household size, source of drinking water, mothers body mass index, multiple birth, birth order, and anemia level of children were found as significantly associated factors for child composite index anthropometric failure and household wealth index. This means that children from poor households are more likely to be underfed, get sick, least likely to access safe water and receive sufficient health-care facilities, and exposed to composite index anthropometric failure. Thus, the authors recommend to concerned bodies and policymakers work on household wealth index to reduce the prevalence of child composite anthropometric failure.

## Data Availability

The datasets for generated analyses during the study is available in Ethiopian Demographic and Health Survey data if unique request sent via their site EDHS 2016.

## Abbreviation

CIAF: Composite index anthropometric index
HHWI: Household wealth index
BMI: Body mass index
EDHS: Ethiopian demographic and health survey
OR: Odds ratio
CCR: Correct classification rate
SNNPR: South Nations Nationalities and Peoples Representatives
